# Focal Therapy: Overcoming Barriers for Advances in Prostate Cancer Treatment in South America

**DOI:** 10.1590/S1677-5538.IBJU.2023.0539

**Published:** 2024-03-18

**Authors:** Becher Ezequiel, Borghi Marcelo, Thomas Polascik, Art Rastineshad, Lara Rodriguez -Sanchez, Rafael Sanchez-Salas

**Affiliations:** 1 Centro de Urología Buenos Aires Argentina Centro de Urología – CDU, Buenos Aires, Argentina; 2 Duke Cancer Institute Durham NC USA Duke Cancer Institute, Durham, NC, USA; 3 Smith Institute for Urology Lenox Hill Northwell Health New York NY USA Smith Institute for Urology, Lenox Hill, Northwell Health. New York, NY, USA; 4 L’Institut Mutualiste Montsouris Department of Urology Paris France Department of Urology, L’Institut Mutualiste Montsouris, Paris, France; 5 McGill University Division of Urology Department of Surgery Montréal Quebec Canada Department of Surgery, Division of Urology. McGill University, Montréal, Quebec, Canada

## INTRODUCTION

Prostate cancer (PCa) is a significant cause of global cancer-related mortality, and South America is no exception (1, 2). Despite advancements in adopting active surveillance for low-risk PCa, which has significantly decreased overtreatment, radical treatments are the only guideline-approved active treatment modalities. The side effect profile of these treatments can considerably affect a patient’s quality of life, leading to high rates of patient-reported regret (3).

Focal therapy (FT) has emerged as a potential alternative to serve as a more balanced option between cancer control and quality of life preservation. FT’s central concept is treating the part of the gland that hosts the clinically significant PCa through a minimally invasive, image-guided procedure (4). The primary goal of this approach is to achieve a more balanced result between treatment efficacy and preservation of genitourinary function.

This editorial explores the barriers impeding the widespread adoption of FT for PCa treatment in South America and presents a successful case of employing this technique on the continent.

### BARRIERS TO FOCAL THERAPY ADOPTION IN SOUTH AMERICA

Despite urologists’ familiarity and acceptance of the FT technique, cost and access to technology represent significant obstacles to widespread adoption of FT. Rigid and uncompromising regulatory processes in some South American countries are the first gate that must be opened.

Brazil is a clear example of this. FT has been labeled as an "experimental treatment" in this country. Sufficient clinical evidence in the body of literature, along with one level I evidence study support the implementation of FT to persuade policymakers to change this designation, thus allowing for a natural evolution of this technique and broader acceptance. FT is no longer an "experimental treatment," and international guidelines (such as the EAU) now allow several institutions to offer FT (with high-intensity focused ultrasound (HIFU) or cryoablation) within prospective registries (5).

In Brazil FT emerged in 2010, starting with HIFU. Since then, 1633 treatments were delivered, however only in the Southwest and South regions of the country, the most socioeconomic developed states. Under private and insured health assistance systems, 446 cases were performed at AC. Camargo Cancer Center (346 for primary tumors and 100 for post-radiotherapy salvage procedures), 25 of these cases being focal treatments. Since 2018, at Israeli Hospital Albert Einstein, 185 treatments have been done, 170 focal HIFU treatments, and more recently, 15 focal Irreversible electroporation procedures. The Moriah Hospital reports 12 patients treated with focal HIFU. In Parana State, 639 HIFU treatments have been performed.

At the Brazilian Public Health System, in Brigadeiro Hospital in São Paulo State, 202 HIFU procedures (143 focal HIFU and 69 whole gland treatments) have been performed. In Rio de Janeiro State University UERJ, since 2017, 78 Focal HIFU have been done including 2 salvage procedures (Table 1). At this point, we are unaware of the actual outcomes accomplished with these operations but it becomes clear that Brazil remains at the forefront of Urological development which happened in the past with prostatic minimally invasive surgery.

**Table 1:** Prostate Cancer Focal Therapy in Brazil.

Institution, City State	Health System	FT Modality/ Equipment (year of starting)	N total	Prymary	Salvage	Focal/ Hemiablation	Whole gland
AC Camargo Cancer Center São Paulo-SP	Insured/ Private	HIFU- Ablaterm ? (2010-2017) HIF Focal One? (2018)	446	346	100	25	321
Hospital Israelita Albert Einstein São Paulo-SP	Insured/private and Public Health System	HIFU Focal One ? (2018) IE -Irreversible electroporation	185 (170 HIFU) and 15 IE)	185	–	185	0
Hospital Moriah São Paulo-SP	Insured/private/	HIFU Focal One ? (2018)	12	12	–	12	0
Hospital Brigadeiro São Paulo-SP	Public Health System	HIFU Focal One ? (2018)	202	143	0	143	69
Hospital Pedro Ernesto- Universidade do Estado do Rio de Janeiro - UERJ Rio de Janeiro-RJ	Public Health System	HIFU Focal One ? (2018)	78	76	02	78	0
Hospital Nossa Senhora das Graças Curitiba -PR	Insured/private/	Sonablate 500 ? (2011)	639	_	–	30% of the cases	80% of the cases
Hospital Santa Catarina São Paulo-SP	Insured/private/	Sonoablate 500 ?	71	–	–	-30% of the cases	80% of the cases

After April 2020, FT for PCa in Brazil has been authorized only in the research protocols scenario. For a case to be done, ethical approval and funds have to be obtained in advance. Such an experience represents the possibility of joining a prospective registry like the one proposed by the Focal Therapy Society (FTS) to reach a clearer understanding of the role of FT in the region. Because FT is in development, it is reasonable to enter outcomes data into a registry, and these registries do exist. NCCN guidelines have endorsed the use of cautious implementation of FT under a registry and also encouraged by the FDA and EMA.

Indeed, if healthcare systems begin to allow the use of FT in clinical practice, the industry will be greatly encouraged to participate. Industry participation is also followed by affordable (and often free) training for physicians, and as the market starts to open and expand, costs will naturally drop.

It is also true that the results of FT heavily rely on good quality multi-parametric MRI (mpMRI). Although significant advances have been made in the field of mpMRI, inter-reader variability is an issue, as is access to high-quality scans, especially in developing countries, this is one of the many reasons PCa diagnosis should still be done with targeting and systematic biopsies (6). However, every day wider acknowledgment of the importance of mpMRI on PCa detection is making this diagnostic tool more accessible, even in lower-income countries.

#### The Example of Argentina

In Argentina, the healthcare regulatory institution (ANMAT) has approved the use of HIFU and cryoablation for PCa treatment. This approval and the non-objection of medical associations have opened the door for FT use in clinical practice. Therefore, institutions interested in FT implementation do have the opportunity to offer this treatment. This has been the case of the Centro de Urología (CDU), a private institution dedicated to Urology that has been the first (and up to now the only) institution in the country to offer FT. The institution has started implementing HIFU as a FT modality since 2009 and thus far has safely done 287 cases. Most cases have been done in the radio recurrent setting, as some insurance companies have been willing to cover this clinical service for these patients. However, a significant (and growing) proportion of patients have also been treated in the primary setting. Oncological, functional, and safety results have mirrored the ones reported in the literature, with 73% of patients avoiding radical treatment (7). The institution has also adopted focal cryoablation since 2021, and results have just been presented at the latest Argentinean Congress (SAU). Thirty-three patients were safely treated, with only one case of Clavien III complication (cystoscopy for hematuria), only 3 cases of recurrence, and no cases of metastasis or mortality.

However, due to Argentina’s long story of import tampering policies, the costs have remained high, and widespread adoption of the technique has not been reached yet. This same obstacle is faced by, for example, robotic surgery, with only four fully working robotic programs (each with only one platform) in the country. Nevertheless, we remain optimistic that when the Brazilian regulations allow for clinical implementation of FT, the wide embracing of the technique will be accompanied by greater interest in the industry to the region and, therefore easier access to the technology.

### MOVING FORWARD WITH FOCAL THERAPY

As previously stated, South America has been at the forefront of minimally invasive prostatic surgery for many years and it does not seem different for the development of FT. In addition to the challenges discussed, it is crucial to underscore the significance of ongoing research, education, and awareness campaigns not only for FT but also for PCa in general. Further studies and clinical trials can provide valuable insights into the effectiveness of FT in different patient populations. Moreover, educating both healthcare professionals and the general public about the benefits and options available in PCa treatment can lead to earlier detection and informed decision-making. By fostering a culture of knowledge and collaboration, we can collectively strive towards a future where PCa patients in South America have access to the best possible care and treatments, aligning with global advancements in urological care. This is also the mission of the FTS, a non-profit academic organization dedicated to improving the field of FT.

## CONCLUSION

Widespread FT adoption in South America faces challenges related to cost, accessibility, and anchored regulatory processes. While select centers excel in providing comprehensive FT, it is essential to integrate FT into the healthcare system to expand access for patients. Urologists, medical associations, regulatory institutions, and industry must work together to promote techniques to better serve our patients. No doubt, overcoming these challenges will pave the way for a brighter future in PCa treatment in South America, aligning with global advancements in urological care.
